# Late Vascular Injury at Both Edges of the VIABAHN Stent Graft after Endovascular Repair for Idiopathic Superficial Femoral Artery Rupture

**DOI:** 10.3400/avd.cr.20-00106

**Published:** 2021-06-25

**Authors:** Yuta Tajima, Kyosuke Kokaguchi

**Affiliations:** 1 Department of Vascular Surgery, Osaki Citizen Hospital, Osaki, Miyagi, Japan

**Keywords:** late vascular injury, stent graft, idiopathic SFA rupture

## Abstract

The VIABAHN stent graft is often used for traumatic and iatrogenic vascular injuries. In this case, vascular injury at both edges of the VIABAHN stent graft was noted 4 months after endovascular repair for idiopathic superficial femoral artery (SFA) rupture. The longitudinal compression of the SFA with a decrease in hematoma size was assumed to exceed the flexibility of the stent graft. Thus, the use of stent grafts for vascular injuries with giant pseudoaneurysms may result in late vascular injuries at both edges of the stent graft. Therefore, cautious assessment of its indications and strict surveillance are required.

## Introduction

The VIABAHN® (W. L. Gore®, Flagstaff, AZ, USA) stent graft has been determined to be effective for the treatment of peripheral artery disease (PAD) in the superficial femoral artery (SFA). Moreover, it is often used for traumatic and iatrogenic arterial injuries due to its minimally invasive property and long-term outcomes.^1,2)^ Its use has been correlated to the risk of late graft occlusion. However, the occurrence of late vascular injury due to the use of a stent graft placed in an appropriate position has been deemed rare.

In this study, we report a case of a patient who presented with late vascular injuries at both edges of the stent graft (VIABAHN) after endovascular treatment for idiopathic SFA rupture with a giant pseudoaneurysm.

## Case Report

A 61-year-old Asian woman was admitted to the emergency room at our institution due to exacerbating pain in the right thigh and swelling 2 days back. Two months prior to admission, the patient reportedly underwent patch-closure surgery for a ruptured sinus of Valsalva and re-surgery for re-rupture of the sinus of Valsalva after 1 month (aortic valve replacement [mechanical valve] and tricuspid valve repair). The patient was taking warfarin after cardiac surgery.

The patient’s level of consciousness was found to be normal, and she was not in a state of shock. Significant swelling and pain in the right thigh were observed. However, no signs of infection were noted, and there was no ischemia in the right lower extremity. Blood examination findings upon admission were as follows: white blood cell count, 7350/µL; C-reactive protein level, 5.23 mg/dL; and procalcitonin level, 0.21 ng/mL. As per her contrast-enhanced computed tomography (CT) scans, a giant pseudoaneurysm (with a diameter of 10 cm) was found in her right thigh with extravasation from the main trunk of the distal SFA. A shift and lumen narrowing of the SFA due to pseudoaneurysm displacement were observed. The three tibial vessels were clearly visible.

**Figure 1:**
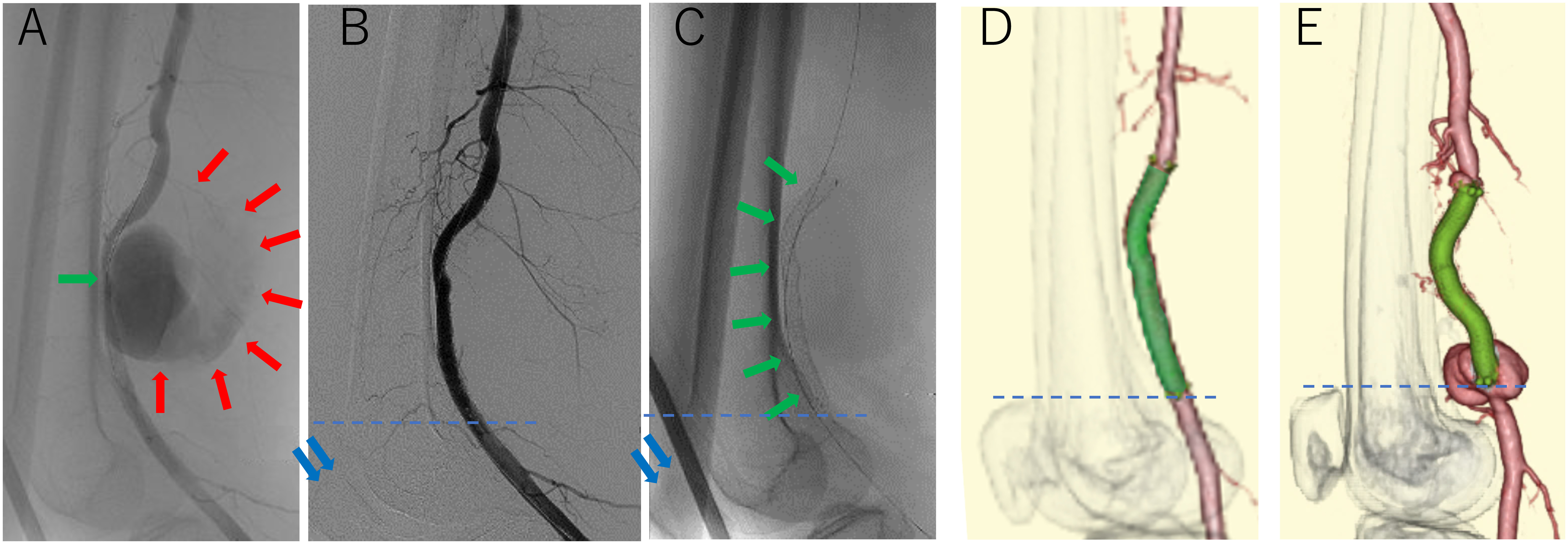
Fig. 1 Chronological changes in the lateral images. (**A**) Digital subtraction angiography (DSA) at the time of rupture. Red arrows, pseudoaneurysm; green arrow, rupture site of the superficial femoral artery (SFA). (**B**) DSA after stent graft insertion. Blue arrows, superior margin of the patella. Blue dotted line, level of the inferior edge of VIABAHN (superior to the patella). (**C**) Fluoroscopic image after stent graft insertion. Green arrows, stent graft. Blue arrows, superior margin of the patella. Blue dotted line, level of the inferior edge of VIABAHN (superior to the patella). (**D**) Volume-rendered (VR) images obtained on postoperative day (POD) 19. Green-shaded areas: stent graft. Disappearance of the pseudoaneurysm and repositioning of the SFA. Blue dotted line, level of the inferior edge of VIABAHN (superior to the supracondylar of the femur). (**E**) VR images obtained on POD 137. Green-shaded areas, stent graft. Significant meandering of the stent graft and pseudoaneurysms at both edges. Blue dotted line, level of the inferior edge of VIABAHN (superior to the supracondylar of the femur).

The patient was then diagnosed with SFA rupture and immediately underwent surgery using the endovascular stent graft approach. A 7-Fr sheath was placed into the right SFA with a right common femoral artery antegrade puncture. A 0.018-in stiff wire was cannulated into the below-the-knee artery, and the VIABAHN (self-expanding type, 8 mm×10 cm) stent graft was placed, without predilatation, from the distal SFA (internal diameter: 7.3 mm) to the above-the-knee popliteal artery (internal diameter: 7.1 mm) superior to the patella (**Figs. 1A**–**1C**). Angiography revealed the successful exclusion of the pseudoaneurysm and good peripheral blood flow without postdilatation. The postoperative course was deemed favorable. Aspirin was administered, and the patient was discharged 13 days after endovascular treatment. CT at 19 days after endovascular treatment revealed that the hematoma size had reduced, and the stent graft was patent (**Fig. 1D**). Ultrasonography at 3 months after the endovascular treatment revealed the same findings.

**Figure 2:**
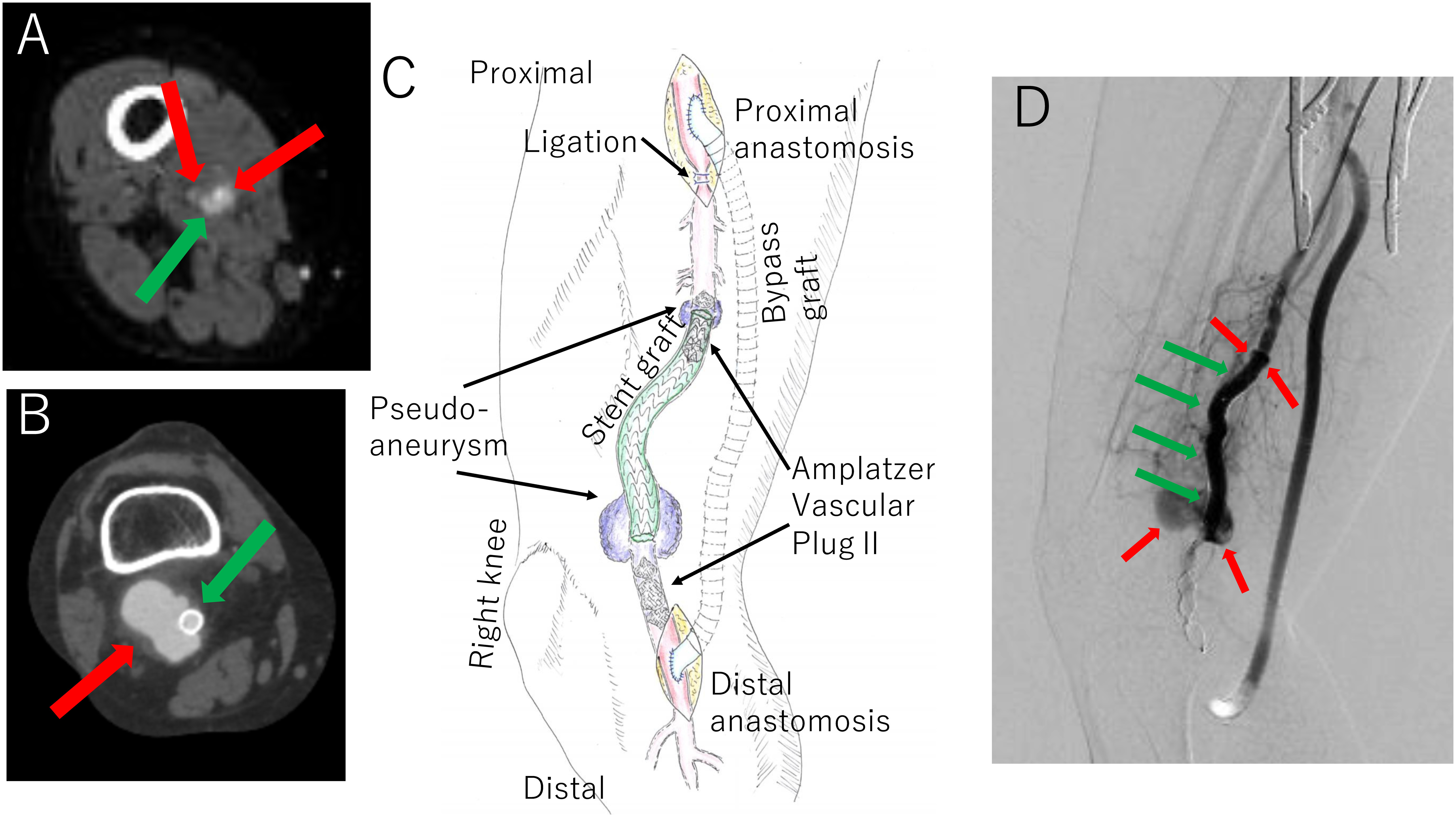
Fig. 2 Computed tomography images obtained on postoperative day 137 and the findings of exclusion and bypass procedures and embolization. (**A**) Axial view of the proximal edge of the stent graft. Green arrow, superficial femoral artery (SFA); red arrows, pseudoaneurysm. (**B**) Axial view of the peripheral edge of the stent graft. Green arrow, SFA with the inserted stent graft; red arrow, pseudoaneurysm. (**C**) Scheme of the surgery. (**D**) Digital subtraction angiography before ligation of the SFA. Green arrows, stent graft; red arrows, pseudoaneurysm.

However, 125 days after endovascular treatment, the patient was admitted to the Department of Cardiovascular Surgery due to congestive heart failure. She experienced pain behind the right knee after hospitalization, and CT at 137 days after endovascular treatment revealed the presence of pseudoaneurysms at both edges of the stent graft (**Figs. 1E**, **2A** and **2B**). There were no findings indicating the presence of an infection or ischemia in the right lower extremity.

The patient underwent emergent hybrid surgery, which comprised the following: prosthetic graft bypass (GORE® PROPATEN® integrated ring, 8 mm) from the middle SFA to the below-the-knee popliteal artery, embolization of the behind-the-knee popliteal artery (Amplatzer Vascular Plug II®, 14 mm), embolization of the proximal edge of the stent graft (Amplatzer Vascular Plug II®, 14 mm), and ligation of the SFA at the peripheral area of the proximal graft anastomosis (**Figs. 2C** and **2D**). The middle SFA and the below-the-knee popliteal artery, the regions of anastomosis, were moderately sclerotic, and pseudoaneurysm thrombosis and good peripheral blood flow were observed. Antibiotics were administered 5 days after the surgery. The patient was discharged after the treatment of congestive heart failure.

Ten months after the lower extremity bypass, the hematoma size was noted to decrease, the bypass was patent, and there were no signs of infection. Moreover, no new vessel ruptures were observed, including that in the area of the anastomosis.

## Discussion

Pseudoaneurysms are often caused by trauma and infections, or they may be iatrogenic.^2,3)^ In some cases, they may be idiopathic or can be caused by diseases, such as Behcet’s disease or congenital connective tissue diseases.^4,5)^ In this case, no history of trauma, treatment, or infections was noted. Behcet’s disease and congenital connective tissue disease were also ruled out based on the patient’s age, physical and blood examination findings, normal aortic valve pathology (appendix), and imaging results. Furthermore, the presence of true aneurysms was also dismissed, as per her CT findings. Therefore, the diagnosis was idiopathic SFA rupture, which is determined to be a rare condition. To the best of our knowledge, only seven other cases presenting with this condition have been reported worldwide.^6)^ The cause of idiopathic SFA rupture remains to be unknown. In young patients, some investigators have reported connective tissue disorder or congenital arterial abnormalities, such as Ehlers–Danlos syndrome, as the possible causes of idiopathic SFA rupture.^6)^ Meanwhile in elderly patients, atherosclerosis is considered to be its primary contributing factor.^6)^ But in this case, systemic atherosclerosis might have caused both rupture of the sinus of Valsalva and SFA.

An appropriate treatment regimen is chosen based on the rupture site, cause of the rupture, presence of an infection, peripheral ischemia, age, and performance status.^3,6,7)^ In this case, an endovascular approach was determined as the most suitable treatment because of the presentation, history of cardiac surgery, intake of warfarin, and presence of a giant pseudoaneurysm. We opted to not select open surgery considering the risks of general anesthesia complications, massive bleeding, and wound infections. Because the rupture hole and pseudoaneurysm were large, coil embolization and thrombin injection were deemed to be inappropriate. Percutaneous insertion of a stent graft was instead selected because there were healthy landing zones, both proximal and peripheral of the rupture site, greater than or equal to 2 cm, which did not come in contact with the joints.

Although there have been a number of reports on late vascular injury after bare stent insertion into SFAs avoiding knee joints, most cases are due to the fracture of the stent body, and not the edges, owing to physiological loading; thus, the deformity and torsion of the distal SFA could be due to muscle movement with knee joint motion.^2)^ There are also reports examining late vascular injuries at stent graft edges after using the VIABAHN stent graft across the joints (non-stenting zone) or using an oversized VIABAHN.^2)^ However, no study has assessed the occurrence of late arterial injuries in patients with PAD using a proper size VIABAHN stent graft for SFA with proper landing zones (healthy vessels greater than or equal to 2 cm), avoiding the hip or knee joints (non-stenting zone), which is attributed to excellent flexibility and the graft material.^8,9)^

In this case, the cause of arterial injuries at the edges of the stent graft might have been the insertion of the stent graft into the SFA in the state of being extended and bended by the giant pseudoaneurysm due to idiopathic SFA rupture. CT scan at 19 days after endovascular treatment revealed a tendency for repositioning and longitudinal compression of SFA due to a decrease in hematoma size, and CT scan after 4 months revealed further repositioning and compression of the SFA and significant meandering of the stent graft (**Fig. 1**). Physiological loading with the knee joint motion might have had some effect on the vascular injury at the distal edge (i.e., near the knee joint), but little effect at the proximal edge (i.e., far from the knee joint). Therefore, excessive loading on both edges of the graft due to the repositioning and longitudinal compression of the SFA with a decrease in hematoma size was assumed to have led in late vascular injury. Such a mechanism of vascular injury can occur in any location when the VIABAHN stent graft is inserted to treat giant pseudoaneurysm and not PAD; no pseudoaneurysms were observed.

The surgical bypass procedure and stent graft reinsertion have been considered for new pseudoaneurysms.^10)^ Recently, endovascular repair using stent grafts across the knee joint was performed for popliteal artery aneurysms. However, the long-term outcomes of placement at the same site are yet to be clearly elucidated, and this treatment option was not selected in this case due to the risk of recurrence of vascular injury at the stent graft edges.^8,9)^ Exclusion and bypass procedures from the SFA to the below-the-knee popliteal artery were selected. However, autovein was not used considering the risk of coronary artery bypass and good runoff below-the-knee artery.

In this patient’s case, the rupture of an infectious aneurysm was not completely ruled out but was thought to be unlikely as there were no findings of an infection throughout the treatment process and also the hematoma quickly disappeared after the exclusion procedure alone, without drainage or long-term use of antibiotics.

Thus, cautious follow-up, including assessments of graft occlusion, infection, anastomotic pseudoaneurysms, and blood vessel rupture at other sites, is necessary.

## Conclusion

As per the findings of this study, it was determined that endovascular treatment using stent grafts for arterial ruptures with giant pseudoaneurysms may result in new arterial injuries at the stent graft edges due to the repositioning and longitudinal compression of the artery associated with the decrease in hematoma size after treatment. Therefore, cautious assessment of indications and strict surveillance are a must.
