# Supplementary material for: Late Vascular Injury at Both Edges of the VIABAHN Stent Graft after Endovascular Repair for Idiopathic Superficial Femoral Artery Rupture
Source: Ann Vasc Dis. 2021 Jun 25;14(2):173–6. doi: 10.3400/avd.cr.20-00106 (PMC8241556; doi:10.3400/avd.cr.20-00106)
Supplement: Supplementary Data [file avd-14-2-cr.20-00106_s001.pdf]

Appendix: Pathological findings of aortic valve (normal pathology)

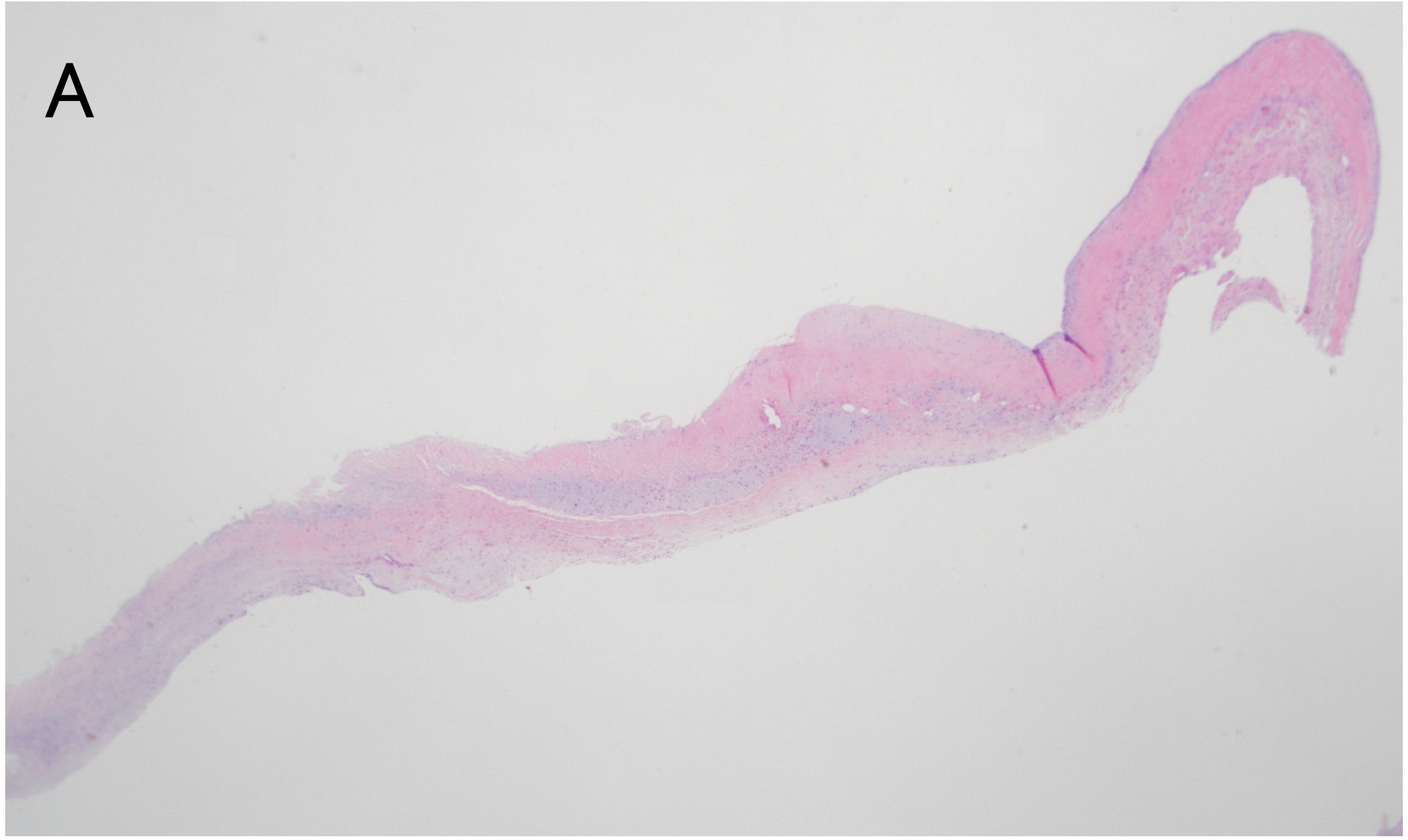

A: Hematoxylin–eosin staining

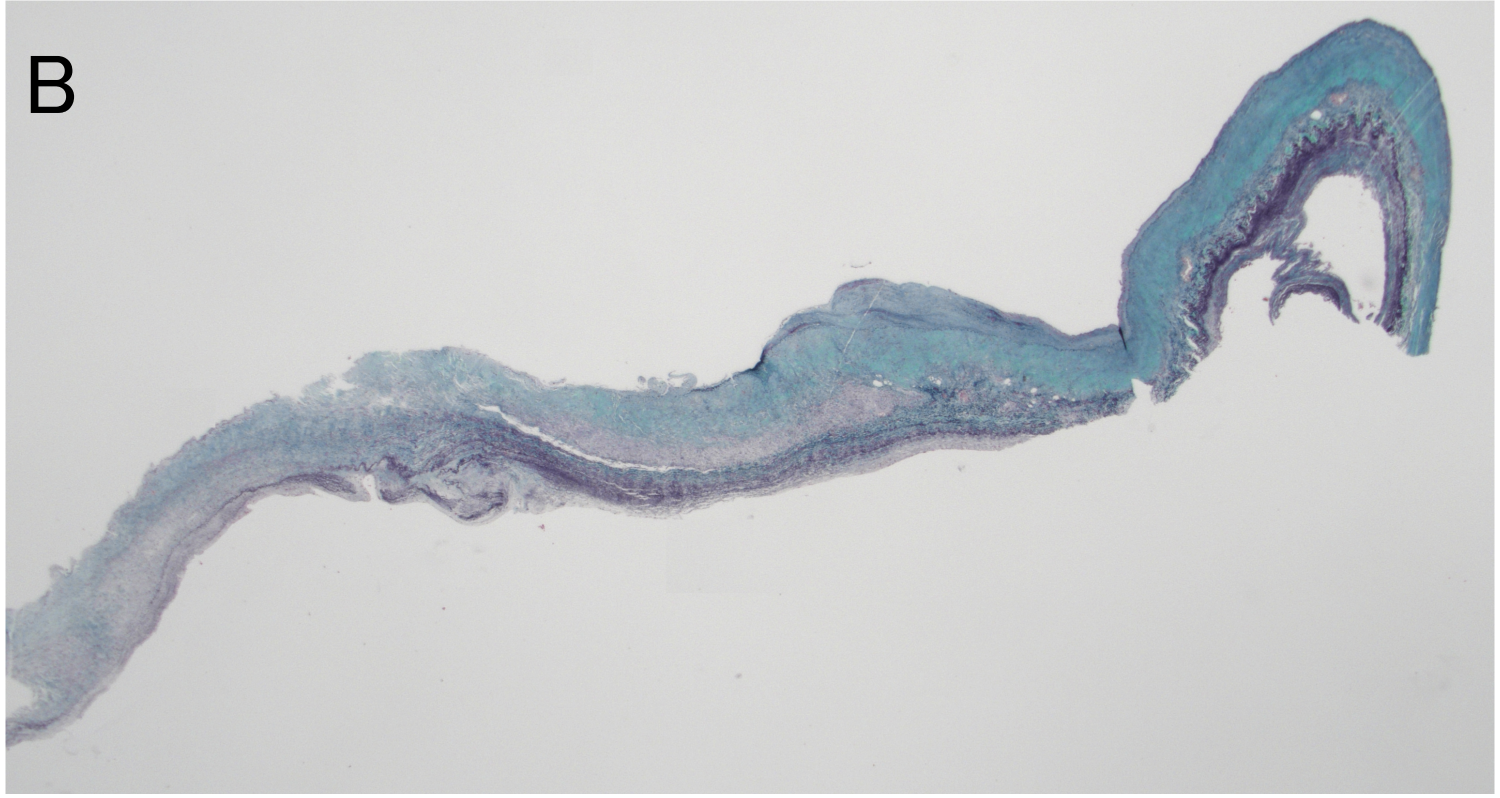

B: Elastica Masson staining
